# Advancements in Soft-Tissue Prosthetics Part A: The Art of Imitating Life

**DOI:** 10.3389/fbioe.2020.00121

**Published:** 2020-03-31

**Authors:** Rena L. J. Cruz, Maureen T. Ross, Sean K. Powell, Maria A. Woodruff

**Affiliations:** Institute of Health and Biomedical Innovation, Queensland University of Technology, Brisbane, QLD, Australia

**Keywords:** prosthetic, prosthesis, polymer, silicone, additive manufacturing, maxillofacial

## Abstract

Physical disfigurement due to congenital defects, trauma, or cancer causes considerable distress and physical impairment for millions of people worldwide; impacting their economic, psychological and social wellbeing. Since 3000 B.C., prosthetic devices have been used to address these issues by restoring both aesthetics and utility to those with disfigurement. Internationally, academic and industry researchers are constantly developing new materials and manufacturing techniques to provide higher quality and lower cost prostheses to those people who need them. New advanced technologies including 3D imaging, modeling, and printing are revolutionizing the way prostheses are now made. These new approaches are disrupting the traditional and manual art form of prosthetic production which are laborious and costly and are being replaced by more precise and quantitative processes which enable the rapid, low cost production of patient-specific prostheses. In this two part review, we provide a comprehensive report of past, present and emerging soft-tissue prosthetic materials and manufacturing techniques. In this review, part A, we examine, historically, the ideal properts of a polymeric material when applied in soft-tissue prosthetics. We also detail new research approaches to target specific tissues which commonly require aesthetic restoration (e.g. ear, nose and eyes) and discuss both traditional and advanced fabrication methods, from hand-crafted impression based approaches to advanced manufactured prosthetics. We discuss the chemistry and related details of most significant synthetic polymers used in soft-tissue prosthetics in Part B. As advanced manufacturing transitions from research into practice, the five millennia history of prosthetics enters a new age of economic, personalized, advanced soft tissue prosthetics and with this comes significantly improved quality of life for the people affected by tissue loss.

## Introduction

Physical disfigurement due to congenital defects, trauma, or cancer causes considerable distress and physical impairment for millions of people worldwide. It impacts their economic, psychological and social wellbeing, often with devastating effects (Tagkalakis and Demiri, 2009). Significantly, physical disfigurement leads to a disruption of body image; an individual’s mental perception of their physical self (Galpin, 1996; Tagkalakis and Demiri, 2009). This fundamental and critical factor of identity affects emotions and influences their decision making. In addition to body image, deformities can lead to bullying, discrimination, and reduced social and economic opportunities.

Since as early as 3000 B.C., prosthetic devices have been used to address these issues and restore both aesthetics and utility to those with disfigurement (Ring, 1991). A prosthetic device is referred to technically as a prosthesis, though it is commonly referred to as a prosthetic. For the purposes of this paper, prosthetic will be used solely as an adjective and prosthetics to refer to the prosthetic field. For optimal prosthetic performance, many factors must be considered such as fabrication methods, aesthetics, function, attachment, robustness, and cost. Fundamental to all of these is the choice of materials.

From basic carved wood in the middle of the last century (Ring, 1991) to current composite 3D printable polymers, materials and fabrication methods in prosthetics have become more sophisticated over time. Improvements in materials science and fabrication engineering, such as 3D printing, will further improve key aspects of prostheses leading to better outcomes. This article is a thorough review of the literature surrounding the applications, chemistry, fabrication processes and physical properties of the most significant polymers used in soft tissue prosthetics, both today and moving into the future.

### History of Prosthetics

**FIGURE 1:**
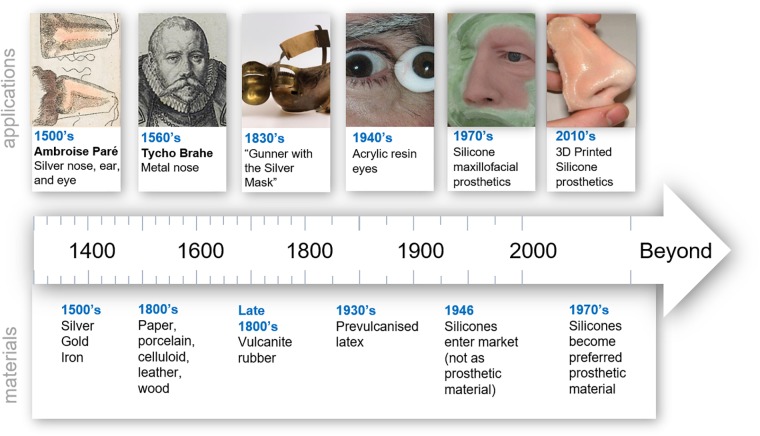
Timeline of trends in prosthetic materials. “Hand colored illustration of a prosthetic nose. 1561 By: Ambroise Paré.” Reproduced under a Creative Commons Attribution 2.0., Tycho Brahe image. Reproduced with permission from “Tycho Brahe Museum,” Gunner with the silver mask. Reproduced with permission from University of Edinburgh, Ocular prosthetic image. Reproduced with permission from Oculuar Prosthethic Inc., Nose prosthesis. Reproduced with permission from JM Yates.

Early prostheses were hand formed out of the most basic natural materials. As materials knowledge improved, more sophisticated material choices became available and led to improvements in quality, durability, biocompatibility, aesthetics, and fabrication approaches. A summary of some key innovations in prosthetics over time is illustrated in Figure 1. In the 16th century prosthetic noses, eyes and palates were crafted from wax, parchment, wood, gold, silver, copper and hard rubber (Ring, 1991). For example, Ring et al. (Ring, 1991) describes a silver prosthetic ear, a nose and eyes made by Ambroise Paré during the 16th century. Metals were a key prosthetic material through to the 19th century with the ability to be shaped and moulded as required (Andres et al., 1992b; Lai and Hodges, 1999). Significant historical events were often a driver in materials innovation. In the 20th century, World War I and II created a large demand for prostheses and reconstruction but there was a limited supply of glass. A substitute came in the way of polymer acrylic resin which quickly became the most common prosthetic material (Artopoulou et al., 2006; Patil et al., 2008). The use of polymers as the main material in the fabrication of prostheses has continued ever since. It wasn’t until the 1960’s that silicones were first used by Barnhart (1960) and became the materials of choice in external soft tissue prosthetics in the 1970s (Gearhart, 1970). Silicones offer many benefits in addition to their ability to mimic soft tissue, such as their ease of manipulation, chemical inertness, durability and strength (Andres et al., 1992b). Today, researchers are making advancements with new prosthetic technology through 3D scanning, 3D modeling and 3D printing, along with modern synthesis of advanced polymeric materials. This generates novel prosthetic solutions that cannot be produced using traditional approaches, and leads to real-world clinical outcomes with a focus on higher patient satisfaction from increased customization and increased accessibility.

#### The Impact of Disfigurement

The psychological adjustment to an acquired disfigurement is challenging for many people, but there are very few studies that provide empirical evidence showing its impact on people’s lives. One limitation of many studies conducted on congenital conditions, is that most have been retrospective, and in many cases, they consist of clinically insignificant participant numbers (Horlock et al., 2005; Steffen et al., 2008, 2010; Tam et al., 2014; Johns et al., 2016). However, the few published studies are informative. A prospective study by Li et al. (2010), which included 170 participants with a congenital malformation of the external ear (termed microtia), observed the psychological effect of this condition as well as the effects of surgical correction. The most significant findings were that psychosocial problems begin at approximately 8 years of age and significantly improve after successful surgical correction. However, a poor reconstructive result was found to result in a negative impact on body image. A study by Noor and Musa (2007) suggested that, in children born with cleft lip and/or palate, between 62 and 75% of participants report experiencing teasing (Hunt et al., 2006). Similarly, in the case of tumor surgery such as mastectomy, the negative impact on body image, sexuality and psychological health is well documented (Maguire et al., 1978; Wolberg et al., 1989; Ganz et al., 1996). However, in these cases it is often hard to distinguish whether these difficulties are due to the surgery or the cancer diagnosis itself (Metcalfe et al., 2004). Given the importance of mental health on the life experience of those with disfigurement, it is not surprising that achieving the highest level of prosthetic realism and function is of great significance.

Although prostheses have wide use in cases of both aesthetic and functional disability, from missing limbs to soft tissue damage, this paper focuses on the application of polymers to restore aesthetics.

### Desirable Properties of Polymeric Prosthetic Materials

**FIGURE 2:**
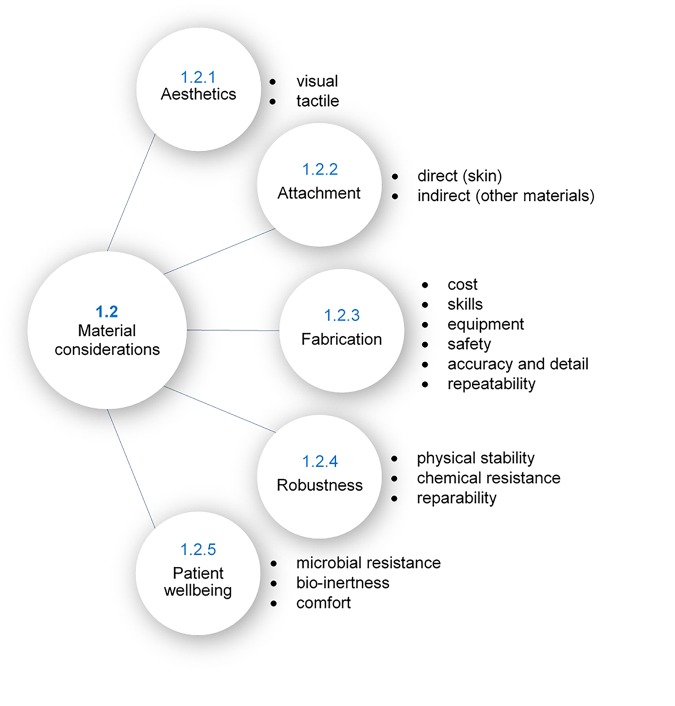
Material specifications in prosthetics.

The desire for both functional and aesthetic prostheses places many unique and often conflicting demands on material selection. To explore this, five core considerations have been found that are discussed in much of the relevant literature, as illustrated in Figure 2; aesthetics, attachment, fabrication, robustness, and patient wellbeing. Achievement of all of these desirable properties is not yet realizable in a single material, however, several existing polymers satisfy many of these requirements. The reader is directed to Part B of this review for details of commonly used materials and their properties.

#### Aesthetics

The visual and tactile properties of a prosthesis are important to ensure it is of the highest realism and is as aesthetically pleasing as possible. This requires the material to be translucent, similar to natural human skin (Bulbulian, 1941; Lewis and Castleberry, 1980; Andres et al., 1992a), and be capable of intrinsic staining to ensure overall color and textures matches the patient’s skin (Lewis and Castleberry, 1980; Andres et al., 1992a). To assist with the homogenous mixing of the colorants, the material must be sufficiently viscous during polymerization (Lewis and Castleberry, 1980). Furthermore, tints must be soluble in the material so as not to clump, and the native color of the material should be neutral to enable effective colorization (Lewis and Castleberry, 1980). Extrinsically, the material must be able to be custom colored to add realistic detailing, preferably without any further modification to the material’s surface (Andres et al., 1992a).

The tactile properties of the prosthesis should also mimic those of skin and flesh to achieve a realistic feel. Skin has a particular softness and pliability when touched, so the material must be soft and with suitable surface elasticity (Bulbulian, 1941; Lewis and Castleberry, 1980; Andres et al., 1992a; Aziz et al., 2003). Lewis and Castleberry (1980) defined the ideal hardness as 25 to 35 Shore A and the ideal tensile strength as 1000 to 2000 psi (6.9 to 13.8 MPa). Surface friction is also important in maintaining a realistic feel, a coefficient of friction of 0.4 to 0.8 is ideal (Lewis and Castleberry, 1980). It is also important to ensure optimal surface tension so the material accommodates oil-based cosmetics for blending (sufficiently low critical surface tension) while still facilitating the removal of the cosmetics with soap and water for cleaning (sufficiently high critical surface tension), defined as 30 to 45 dynes/cm (Lewis and Castleberry, 1980).

#### Attachment

In addition to visual and tactile realism, a successful prosthesis must remain attached to the patient throughout the course of the day as they undergo their usual activities, either by direct adhesion to skin or by mechanical means (e.g. clips or magnets) (Lewis and Castleberry, 1980; Andres et al., 1992a; Polyzois et al., 2000). Direct adhesion to skin requires the chosen adhesive to be compatible with the prosthetic material; achieving adherence without causing material deterioration. The prosthesis, and therefore materials, must also be able to be used and removed without harming the patient or damaging the prosthesis (Lewis and Castleberry, 1980; Andres et al., 1992a; Polyzois et al., 2000). Often mechanical attachment methods require the prosthesis to have an integrated stiff framework. This can be achieved using internal integration of the framework within the prosthetic material and/or by bonding of the framework to the prosthetic material (Lewis and Castleberry, 1980; Andres et al., 1992a). Approaches to reduce the stresses of attachment include limiting the weight of the prosthesis (Bulbulian, 1941; Lewis and Castleberry, 1980; Andres et al., 1992a; Liu et al., 2013), limiting heat conduction to prevent contraction (Bulbulian, 1941), and ensuring sufficient elasticity to enable facial motions and other external forces, depending on the specific requirements (Bulbulian, 1941).

#### Fabrication

The fabrication process of a prosthesis dictates the cost of the prosthesis, repeatability, accuracy and level of detail that can be achieved, as well as fabrication time. Considerations regarding fabrication involve the cost of purchasing and running the required machinery, complexity of methods, cost of materials (prosthetic and otherwise), the safe processing and handling of the materials, storage requirements, shelf life, and the amount of waste produced (Bulbulian, 1941; Lewis and Castleberry, 1980; Andres et al., 1992a; Lai and Hodges, 1999). For example, one consideration is the processing temperature of the material. Some prosthetic materials can be processed at room temperature using low cost dental stone, acrylic or epoxy molds (Lewis and Castleberry, 1980; Andres et al., 1992a; Lai and Hodges, 1999), while others set in higher temperatures, requiring the use of more expensive metal molds with more complex fabrication techniques (Andres et al., 1992a; Lai and Hodges, 1999).

The level of technical skill required to process a given material, as well as the complexity of the required machinery, directly impacts the processing time and cost of the prosthesis. Many polymerization processes, for example, are highly sensitive to both technique and the processing environment (e.g. presence of moisture) (Goldberg et al., 1978; Craig et al., 1980; Aggarwal et al., 2016). This often requires highly skilled technicians so as to avoid failed attempts and waste. It is also important that technicians have sufficient time to work with (i.e. mix, pigment, degas, mold, etc.) the material during polymerization (working time). In addition, the material should be safe to work with and have no toxic by-products or other components that could harm the technician (Andres et al., 1992a).

Fabrication processes directly impact the repeatability and level of detail of the final prosthesis. In the case of molding approaches, the viscosity of the fluid during the working time should be sufficiently low to allow polymer to flow into the narrow parts of the mold (Bulbulian, 1941; Lewis and Castleberry, 1980; Andres et al., 1992a) and achieve that desired detail, while at the same time being sufficiently high to keep any added colorants homogenously suspended in the mixture for consistent coloration (10 000 to 75 000 centipoise or millipascal seconds) (Lewis and Castleberry, 1980). Other considerations include the ease of prosthesis removal from the mold and material shrinkage during processing to preserve detail and ensure the best patient fit (Bulbulian, 1941; Lewis and Castleberry, 1980; Andres et al., 1992a). Shrinking can occur both due to material properties and due to the presence of air in the mixture (Lewis and Castleberry, 1980). Most importantly, the methods should be repeatable to produce consistent high quality prostheses (Bulbulian, 1941; Lewis and Castleberry, 1980; Andres et al., 1992a; Polyzois et al., 2000).

#### Robustness

It is desirable that the properties of a prosthetic material are maintained throughout its service life. Its chemical and physical stability must survive exposure to various environmental conditions [e.g. sunlight (UV), hot or cold temperatures, humid or dry conditions, etc.] (Bulbulian, 1941; Lewis and Castleberry, 1980; Polyzois et al., 2000). Materials can be sensitive to these environmental factors and undergo changes to both appearance and mechanical properties. For example, stiffening of a material can occur at 10 to 20°C above its glass transition temperature (Lewis and Castleberry, 1980). This means the chosen material should have a glass transition temperature sufficiently low to maintain flexibility in cold environments. Furthermore, the heat distortion temperature should be sufficiently high (∼121°C) to prevent distortion during sterilization with boiling water or steam (Lewis and Castleberry, 1980).

It is also preferable for the material to be non-porous, resistant to staining, and therefore washable (Bulbulian, 1941; Polyzois et al., 2000; Aziz et al., 2003). This is important as during washing and regular use, prosthetic materials may be exposed to water, saliva, sweat, and other fluids (Polyzois et al., 2000; Aziz et al., 2003). If absorbed, these fluids might affect physical properties, cause color changes, and cause degeneration of the polymeric structure (Polyzois et al., 2000; Aziz et al., 2003). Furthermore, exposure to fluids is an avenue by which plasticizers and additives may leach out of materials, causing further changes to their physical properties and appearance (Lewis and Castleberry, 1980).

These issues are particularly important with the thin margins at the edges of prostheses which are made to blend with the skin, as they are susceptible to tearing. To prevent this damage, the material requires high tear strength, high tensile strength and high elongation at break (Lewis and Castleberry, 1980; Andres et al., 1992a; Aziz et al., 2003; Liu et al., 2013).

Although primary material robustness is important, it is possible with some materials that adjustment, repair or relining can be performed to extend the service life of the prosthesis (Lewis and Castleberry, 1980; Andres et al., 1992a).

#### Patient Wellbeing

Prostheses are often worn by patients for many hours each day, such that there are several important comfort and tissue compatibility issues that must be considered when selecting a material (Bulbulian, 1941; Lewis and Castleberry, 1980; Andres et al., 1992a). These requirements demand that the prosthetic material should be light weight, not conduct excessive heat, and have sufficient elasticity for tissue material compliance (Bulbulian, 1941; Lewis and Castleberry, 1980; Andres et al., 1992a; Aziz et al., 2003; Liu et al., 2013) so as to reduce stresses on the patient’s tissues. Additionally, the material should be breathable to prevent skin irritation and odorless (Andres et al., 1992b).

**FIGURE 3:**
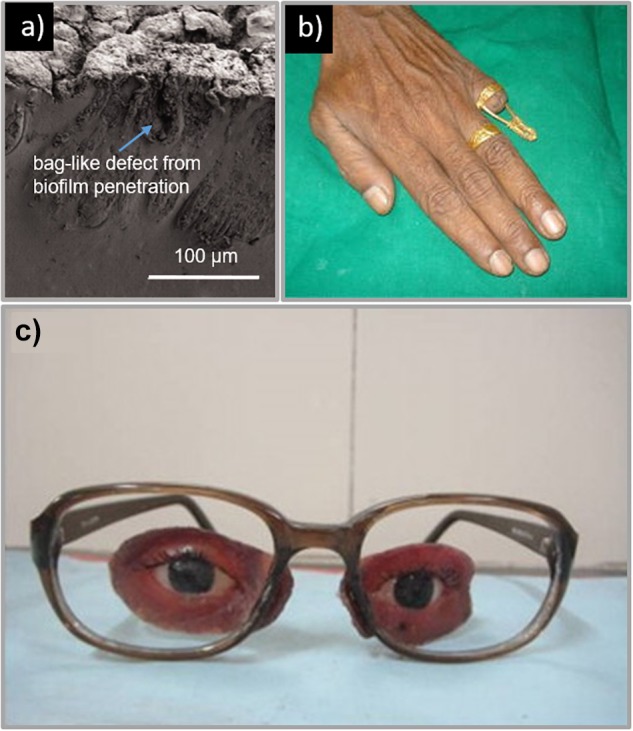
**(a)** Example of a facial prosthesis with sampling side cut made at the margin that is in contact with the skin. The prosthesis shows a tear on the periphery and brownish decoloration where the material is in contact with the skin. Reproduced with permission from Taylor & Francis (Ariani et al., 2012), **(b)** Prosthetic finger; (left) brass rod connected to a ring for attachment of hollow silicone prosthesis. Reproduced with permission from *Springer* (Saxena et al., 2014). **(c)** Facial prosthesis retained by attachment to spectacles. Reproduced with permission from *Elsevier* (Pruthi and Jain, 2013).

The prosthetic material must also have sufficient surface wettability (Andres et al., 1992a; Aziz et al., 2003; Liu et al., 2013) as poor surface wettability leads to poor lubrication of the prosthetic surface. This leads to friction on the skin and thus skin irritation and even infection (Waters et al., 1999; Aziz et al., 2003; Preoteasa et al., 2011; Liu et al., 2013). Poor surface wettability is also correlated with the attachment of microorganisms such as Candida albicans (Park et al., 2003; Frade and Arthington-Skaggs, 2011; Ariani et al., 2012; Li et al., 2012). This commensal microorganism is found in the oral cavity and known to adhere to one another to form biofilms, thereby resisting disinfection (Ariani et al., 2012; Shinde et al., 2012; Atay et al., 2013). The effects of this can be seen in Figure 3a. The formation of a biofilm is not only a nuisance for those trying to keep their prostheses clean, but the microorganisms can also penetrate into the prosthetic material itself leading to bag-like defects (Ariani et al., 2012). This is particularly an issue with prostheses due to the humidity and temperature at the skin-prosthetic interface, a perfect environment for the proliferation of opportunistic bacteria and fungi (Goiato et al., 2010; Ariani et al., 2012).

A study by Ariani et al. (2012) found that skin occluded by silicone prostheses showed ten times more culturable microflora than corresponding unaffected skin. Porosity and roughness have also been shown to play important roles in microbial colonization as they provide pathways into the material and facilitate the spread of unwanted microorganisms (Fernandes et al., 2010; Li et al., 2012; Atay et al., 2013). Fernandes et al. (2010) found that the critical surface roughness is 0.2 μm, below which there is not significant reduction in microbial settling. This presents an issue, as many prostheses are processed using dental stone molds which produce rough surfaces for the colonization of microflora (Hulterström et al., 2008; Atay et al., 2013). One way to control microbial colonization is to ensure that prosthetic materials are able to be easily and thoroughly cleaned (Bulbulian, 1941; Lewis and Castleberry, 1980; Polyzois et al., 2000; Aziz et al., 2003). However, while mechanical methods of cleaning are sufficient in removing biofilms on prosthetic surfaces, they are not able to remove microbes buried within the material (Goiato et al., 2010; Ariani et al., 2012); requiring chemical soaking for sufficient disinfection (Goiato et al., 2010). Therefore the material must also be compatible with these chemical agents; including hypochlorites, peroxides, neutral peroxides with enzymes, acid enzymes, and disinfectants (Goiato et al., 2010).

Given today’s materials and surgical procedures, infections are uncommon (Mohan et al., 2016). However, serious complications can lead to significant consequences for the patient, although there exist only a few studies in literature that discuss the management and treatment of infections related to soft tissue prostheses. Often, in the case of prosthetics, complications are related to the attachment method rather than the prosthetic itself. For example, a study by Hamming et al. (2009) found that in the case of osseointegrated titanium screws used to attach prosthetic ears, no implants failed, 1/3 of the ears developed abutment site skin complications and 1/9 needed soft tissue revision surgery (Hamming et al., 2009). Another more recent study by Al Kadah et al. (2018) found that 71.4% of patients who received osseointegrated silicone prosthetic ears presented with reactions of the skin surrounding the titanium implant site (Al Kadah et al., 2018). Similarly with facial prostheses, issues surrounding the attachment method have been observed. A retrospective study by Karakoca et al. (2010) evaluated 25 orbital and 13 nasal prostheses and found an estimated mean survival time of 14.5 months for the first of the patient’s prosthesis, with common complications involving clip activation, bar screw and abutment loosening and detaching of the silicone from the acrylic substructure (Karakoca et al., 2010). A larger retrospective study involving 99 patients with custom facial prostheses was carried out by Papaspyrou et al. in 2018 (Papaspyrou et al., 2018). This included 53 patients with ear prostheses, 27 with eye prostheses and 19 with nasal prostheses with 82.8% or the prostheses designed to be magnetically attached via osseointegrated implants. The study found no serious complications and no osteoradionecrosis, but found 32% of the patients had skin redness, 17% with itching and 8% suffering burning sensation. In the case of breast implants, the rate of complications is relatively low. A retrospective analysis undertaken in the United Kingdom of 3002 women who received aesthetic breast prosthetics found infections were experienced by 33 patients (1.1%) (Araco et al., 2007). Pittet et al. (2005) found in their global survey of 10914 patients, a similar rate of 1.7% reported acute post-operative infection.

It is also vital that the chosen prosthetic material is bioinert and biocompatible for its intended application and is therefore non-toxic, non-allergenic, and non-carcinogenic (Lewis and Castleberry, 1980; Andres et al., 1992a; Liu et al., 2013).

## Applications of Polymers in External Prosthetics

**FIGURE 4:**
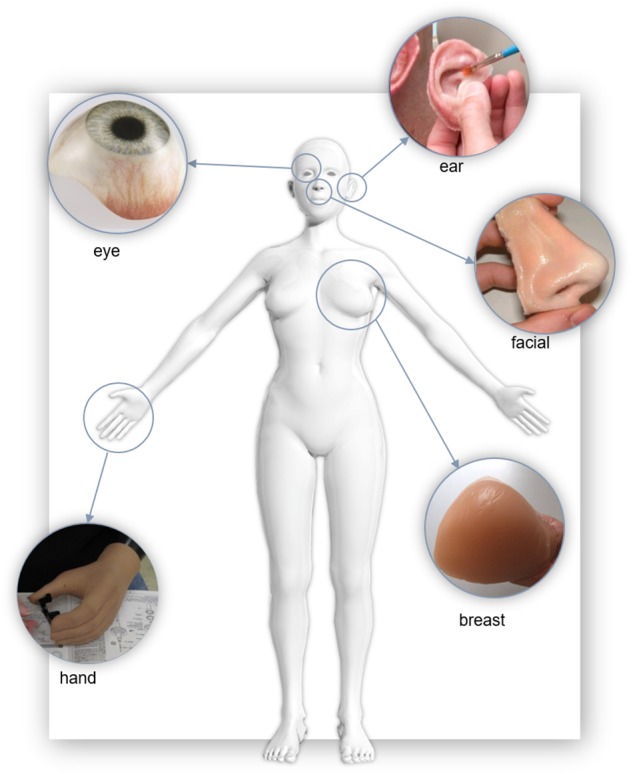
Examples of polymer soft tissue prostheses. “Eye” Reproduced with permissions from *Erickson Labs Northwest* (Northwest_Eye_Design, 2019), “Ear” by United States Air Force photo/Staff Sgt. Kevin Iinuma, “Nose” reproduced with permission from JM Yates (Zardawi et al., 2015a), “Breast” reproduced with permission from the Cancer Australia 2019, “Hand” United States Defense Dept. photo by Fred W. Baker III.

Polymers have found extensive use in modern prosthetics. Here we briefly summarize some important research in the production of prostheses for tissue that commonly requires aesthetic restoration, as illustrated in Figure 4; the ear, face, eye, breast, and hand.

### Prosthetic Ears

Microtia and Treacher Collins syndrome are examples of congenital disorders that result in malformations of the external ear (auricle). The ear may also be lost through trauma or cancer. Although traditional hand-made approaches comprise the majority of prosthetic ears that are fabricated today (Butler et al., 2000; Singh et al., 2013), current research seeks to leverage automated 3D scanning, modeling and 3D printing to create customized, patient specific ear prostheses.

As with prostheses for other regions of the body, traditional fabrication approaches involve taking an impression of existing structures (areas of attachment and anatomy to be replicated such as bilateral structures), casting the existing structures and sculpting the prosthesis, creating the mold for the prosthesis and casting the final prosthesis. For ears, this is often followed up by hand-painting fine details to provide even greater realism. A detailed discussion of traditional fabrication methodology can be found in section “Traditional Approaches.”

One significant advantage of 3D digital approaches over traditional means is that patient anatomy can be obtained without the need for taking uncomforable alginated gel or plaster impressions of the ear. Subburaj et al. (2007) presented methodology for using CT images with CAD/CAM technology to create a prototype prosthesis which was then 3D printed. This work was followed up by Karatas et al. (2011) (Karatas et al., 2011), who used similar methods but included two clinical patient cases. A different approach was taken by Ciocca et al. (2007, 2010a) where a laser scanner was used to capture the patient’s microtia affected ear, with the unaffected ear scanned for use as a model. Using these computer models, they manufactured prosthetic ears by 3D printing an inverse mold of the ear model, which was directly filled with silicone. Liacouras et al. (2011) have adopted a similar approach, utilizing 3D photography systems (3dMD LLC). Section “Impression” further expands on these 3D digital approaches.

### Facial Prostheses

Facial prostheses can include the nose, jaw, and/or surrounding tissue areas. The highly visible and personalized nature of the face makes creating accurate and life-like prostheses both extremely important and challenging. Polymers used in this region must closely match the patient’s skin tone and texture in order to blend in, and contain excellent mechanical properties to ensure robustness and flexibility; particularly in mobile regions around the mouth and jaw. Facial prostheses also often require complex substrates and supporting structures for successful attachment (Kurunmäki et al., 2008). The majority of facial prostheses use medical grade silicone for the bulk of the prosthesis, although direct 3D printed starch infiltrated with silicone has also been explored (Xiao et al., 2013, 2014; Zardawi et al., 2015a, b). Aside from matching patient surface color and texture, a central challenge of fabricating facial prostheses is long-term adherence.

In many cases, osseointegrated implants (Fantini et al., 2013) and surgical adhesives have be employed to attach the facial prosthesis to the patient. Other approaches involve attaching facial prostheses to spectacle frames which are then be worn by the patient (Ciocca et al., 2009, 2010b,c, 2016; Bhandari et al., 2014; Neto et al., 2015). In another study involving a larger facial and jaw prosthesis, prostheses were retained using magnets and an acrylic conformer (hollow cylinder) (Venugopalan et al., 2014).

Studies comparing 3D printing of facial prostheses to traditional methods have highlighted a reduced cost and time from using these technologies over more manual and traditional processes (Sansoni et al., 2009; Eggbeer et al., 2012; Sun et al., 2013; Ciocca et al., 2016). In many cases, a digital database was used to pick a nose to best match the aesthetic of the patient’s face (Wu et al., 2008; Ciocca et al., 2010b, c; Qiu et al., 2011; Sun et al., 2013). This model was then smoothed onto the patient’s scan to create a prosthetic design.

In recent studies, Sansoni et al. (2009) and Sun et al. (2011) both 3D printed positive models of the patients face and their prosthetic 3D design. These models were then used to mold wax patterns, and conventional fabrication was used to create the final prosthesis. Substructures were also designed and fabricated using 3D modeling and printers to give the prosthesis stability and provide attachment points. These substructures were put inside the molds before they were packed with either silicone or resin and then polymerized. Ciocca et al. (2009, 2010a) used silicone adhesive to seal the extrinsic colors of the prosthesis which was then finished with a matting dispersion liquid to reduce the gloss of the final facial prosthesis. The most recent clinical report by Ciocca et al. (2016), building on their earlier work using 3D scanning and printing for fabricating a prosthetic nose, described that reducing the minimum thickness of peripheral facial regions to 1.7 mm reduced its weight and created a more skin like appearance and feel. This allowed it to more accurately follow facial movements when speaking and smiling.

A study by Eggbeer et al. (2012) compared two different 3D printing techniques for fabricating prosthetic noses; a direct and an indirect approach. The direct approach printed the “body” of the prosthesis in a transparent, acrylate-base material (TangoPlus) using the Objet Connex 500 (Stratasys, Ltd., Eden Prairie, MN, United States) 3D printer. The “TangoPlus” material was chosen because of its similarity to silicone rubber. To complete the prosthesis, a high consistency HC20 silicone (Technovent Ltd., Newport, United Kingdom) was mill rolled to ∼0.4 mm and then wrapped around the base of the prosthesis where a primer was coated to create adhesion between the two materials. A second, viscous layer of HC20 silicone was then wrapped around the prosthesis to create a deeper and more natural color. The indirect approach for fabricating the prosthetic nose used a 3D printer to produce an inverse mold which was then filled with intrinsically stained silicone. The study compared the tensile, elongation and tear strengthen properties of the two prostheses. Despite the promise of direct 3D printing facial prostheses, the results highlighted the limitation of the TangoPlus material for this application. Despite its similar flexibility to conventional silicone, the study found its tensile strength and tear strength limits would result in early failure from daily wear and tear.

### Facial Prostheses of the Eye

Prosthetic eyes can be broadly separated into two types; those for patients with a functioning eye socket and those for patients without. For those with intact eye sockets, only an eyeball prosthesis is required. Otherwise, a customized prosthesis encompassing surrounding soft tissue regions is needed to fully restore the aesthetics of the area. Due to the different mechanical and aesthetic properties between the eye and surrounding tissue, prostheses are often made from multiple materials including acrylic resin, polyurethane and silicone. Attachment of the prosthetic eye depends on the patient case and include the use of the native eye socket, medical adhesive, spectacle frames or osseointegrated implants.

Patients who require only a prosthesis of the eyeball often have the option of stock prostheses. These are mass-produced and are available in a very limited number of sizes and a small range of colors. Whilst cheaper and more accessible, they have the potential to cause irritation due to imperfect size matching. These prostheses also produce voids between the prosthesis and the tissue which collect mucous and debris, potentially leading to infection. These issues are mitigated for customized prostheses that fit well against the tissue bed of the eye socket (Sarin et al., 2015). For example, a case study for the fabrication of a custom prosthetic eye to fit into a functioning eye socket was performed by Gunaseelaraj et al. (2012). In this case, a patient was using a stock prosthesis but had been experiencing irritation due to poor fit. An impression of the eye socket was first obtained using the external tray technique; involving the injection of the ophthalmic alginate impression material through the hollow stem of the impression device (tray) which is held up against the eye socket. Although alginate was used in this study, other impression materials are also available including irreversible hydrocolloid (Sarin et al., 2015), light viscosity polyvinyl siloxane (Shankaran et al., 2016) or silicone based impression materials (Cevik et al., 2012). After the impression was cast, a wax pattern of the prosthesis was made, tried on the patient, and further sculpted to fit the socket. The wax pattern was then used to make a mold which was then filled with scleral resin. Aesthetic details (irises, pupils, fine red threads added to mimic blood vessels) were then painted on. A thin layer of clear acrylic syrup was coated onto the sclera to keep the painting and blood vessels in place. Clear acrylic was then processed onto the sclera and, before final insertion, the prosthesis was disinfected. A similar study by Sarin et al. (2015) described the use of a printed photograph of the contralateral eye in place of hand-painting the blank.

In cases where the eye socket is not functional, the prosthesis is more complex, involving composite materials each having different functions and material properties (Padmanabhan et al., 2012; Veerareddy et al., 2012; Pruthi and Jain, 2013; Shetty et al., 2016). An example of a traditional fabrication approach, described by Shetty et al. (2016), a custom prosthesis made for a patient with facial disfigurement, including the loss of the left eye. This patient did not have any bony or soft tissue underlay to aid attachment of the prosthesis and a two way silicone adhesive was chosen to attach the prosthesis to the skin. The patient chose to use a common attachment approach where the prosthesis is attached to spectacles, similar to other studies (Padmanabhan et al., 2012; Veerareddy et al., 2012; Pruthi and Jain, 2013). These other methods, however, use a heat cured acrylic resin for the soft tissue substitute, which is a more robust material for attaching to spectacles (Padmanabhan et al., 2012; Veerareddy et al., 2012; Pruthi and Jain, 2013), as depicted in Figure 3c. Regardless of the attachment approach, many studies suggest that wearing spectacles can improve the overall cosmetic appearance of the prosthesis (Pruthi and Jain, 2013; Shetty et al., 2016).

As an alternative to traditional approaches, Ruiters et al. (2016) described a fabrication method that used advanced manufacturing. Using CT scans of the patient’s orbital cavity, a prototype prosthetic eye was designed on computer software and 3D printed in resin using the Objet Connex350 3D printer (Stratasys, 2019). This prototype was then trialed in the patient before a final prosthesis was made from acrylic resin in the traditional manner. Another advanced manufacturing approach by Ciocca and Scotti (2014) aimed to restore the aesthetic of the eye and face. Using MRI scans and 3D laser scans of the patient, the patient anatomy was obtained. CAD software was then used to design the final prosthesis. The facial prosthesis required an underlying substructure (polyamide material) which connected the nose piece to the middle of the glasses arm, and to retain the ocular shell. The inverse mold for fabricating the silicone substitute for soft tissue was 3D printed using laser sintering of polyamide powder. The mold was then filled with intrinsically colored silicone followed by post-processing with extrinsic colors, sealants and matting agents. This approach was similar to that used in other clinical case studies by the same author ([citeskum]BR30,BR26,BR27,BR28,BR29,BR33[citeekum]Ciocca et al., 2007, 2009, 2010a,b,c, 2016; Fantini et al., 2013).

### Prosthetic Hands and Fingers

Prostheses for the hand and finger include both non-functional aesthetic prostheses and, more recently, functional prostheses incorporating robotic or bionic electronic components surrounded by a life-like aesthetic outer shell; a cosmetic glove. External materials for these prostheses include both PVC and silicone, which may encase the electronics, being functionally robust and aesthetically pleasing. Investigations have been made into the effect of these cosmetic gloves on the power required from articulating prosthetic hands (Tolou et al., 2012; Smit and Plettenburg, 2013; Kuret et al., 2016), one such study termed this effect as “stiffness compensation” (Tolou et al., 2012).

The majority of case reports describing purely aesthetic prostheses involve the restoration of one or two fingers. The typical fabrication methodology used irreversible hydrocolloid to take an impression of the defect, followed by the creation of a positive cast using dental stone. The final wax patterns were then sculpted using either an impression of the contralateral finger (Shanmuganathan et al., 2011; Jacob et al., 2012; Aydin et al., 2013; Raghu et al., 2013; Saxena et al., 2014) or a donor finger (Arora et al., 2011; Kaira and Dabral, 2014) for their shape. Attachment strategies for non-functional prosthetic fingers include a glove fit over the stump of the remaining finger (Arora et al., 2011; Shanmuganathan et al., 2011; Jacob et al., 2012; Kaira and Dabral, 2014), implants (Aydin et al., 2013), or by a ring (Arora et al., 2011; Raghu et al., 2013). In the case of the glove fit approach, the diameter of the wax pattern was reduced by 0.5–1 mm so the final silicone prosthesis would have to be stretched over the stump, creating a tight fit (Arora et al., 2011; Shanmuganathan et al., 2011; Jacob et al., 2012; Kaira and Dabral, 2014). For the final prosthetic finger, the most commonly used material was RTV silicone. In some cases a thickener was added to the silicone base to give the prostheses a more natural appearance and feel (Jacob et al., 2012; Raghu et al., 2013). Prosthetic fingernails typically employ a heat cured clear acrylic material further secured with RTV silicone or a cyanoacrylate adhesive (Arora et al., 2011; Kaira and Dabral, 2014).

Saxena et al. (2014) developed a separate prosthetic finger fabrication and attachment approach as depicted in Figure 3b. In this case, the wax pattern of the finger was designed to be hollow to allow for the insertion of a conically shaped brass rod (5 mm thick) to be inside the prosthesis for stability. The top of this rod was then interlocked with the silicone prosthesis using a wire mesh welded to the top of the rod. The final prosthetic finger was then attached to the stump of the patient’s finger by connecting the brass rod with a ring at the base of the prosthesis. This was connected to another ring worn by the patient on their intact ring finger to ensure attachment and stability (Saxena et al., 2014).

### Prosthetic Breasts

For women who have undergone mastectomy, restoration of the breast tissue can be vital to their quality of life. In cases where the patient cannot or does not want reconstructive surgery, externally worn prosthetic breasts, typically an off the shelf product, can be used (Glaus and Carlson, 2009; Jetha et al., 2017). Important factors that need to be considered in the design of these prostheses are: how they feel and act comparatively to natural breasts, weight, interaction with scar tissue, and how they will be retained. The weight of prosthetic breasts is also important because of their effect on balance and posture, and the damage they cause to the shoulders and back (Rostkowska et al., 2006; Gallagher et al., 2009).

The most common material used in external prosthetic breasts is silicone gel due to its ability to mimic the feel of a natural breast (Gallagher et al., 2009). These prostheses are usually retained in a brassiere, although self-supporting prostheses are also available. One of the disadvantages of using silicone gel for prosthetic breasts, however, is their weight (Gallagher et al., 2009). Because of this, there is some research around alternative designs for light-weight prostheses, with several patented. In some cases, polyurethane film was chosen as the material for the outer skin layer (Huang, 2009; Laghi and Vint, 2012). For example, Huang (2009) designed a prosthetic breast with a two chamber design underneath the outer polyurethane layer. One chamber contained silicone gel to ensure the prosthesis maintained the ideal feel, and the second chamber contained a lighter substance such as air, liquid or a foamed material to reduce the overall weight of the prosthesis. Another approach taken by Laghi and Vint (2012) replaced the use of silicone gel with a co-polymer gel filler comprising mineral oil, thermoplastic and glass microspheres. This enabled the clinician to heat the prosthesis and remold it for patient customization, unlike silicone prosthetic breasts which have a permanent shape determined by the mold used for manufacturing (Laghi and Vint, 2012).

The use of CAD/CAM technology for the fabrication of personalized prosthetic breasts is described in Eggbeer and Evans (2011). Similar to their approach used for the production of a personalized prosthetic nose (Eggbeer et al., 2012), the authors used a 3DMD 3D scanning system (3dMD LLC) to capture the torso of the patient post-mastectomy. To produce the computer model of the final prosthesis, a scan of the contralateral breast was mirrored. From this model, a two-part mold was designed with two holes – one for injection and the second as a vent. This mold was then 3D printed in clear resin using stereolithography. The advantages of using stereolithography for the mold was the ability to print the mold in a translucent material so they could ensure the mold was filled. To reduce the weight of the prosthesis from that of a silicone-gel-only prosthesis, a low-density, open-cell foam polyurethane was molded for the center of the prosthesis which was then surrounded by silicone.

Given the range of available sizes, shapes, weights and colors, the use of off-the-shelf prosthetic breasts provide many options for women post-mastectomy (Gallagher et al., 2009; Glaus and Carlson, 2009). With the rise in 3D printing for fabricating prostheses, the ability to produce personalized prosthetic breasts matching the patients’ natural breasts will become widely available.

## Fabrication Methods for External Prosthetics

### Traditional Approaches

Many different prostheses are traditionally fashioned by highly skilled prosthetic technicians, also known as anaplastologists or prosthetists (Larson, 2014). The technicians build up the prostheses over several steps, beginning with taking an impression of the relevant anatomy, followed by sculpting and molding, with the prosthesis cast in the mold before detail is added (Larson, 2014).

#### Impression

**FIGURE 5:**
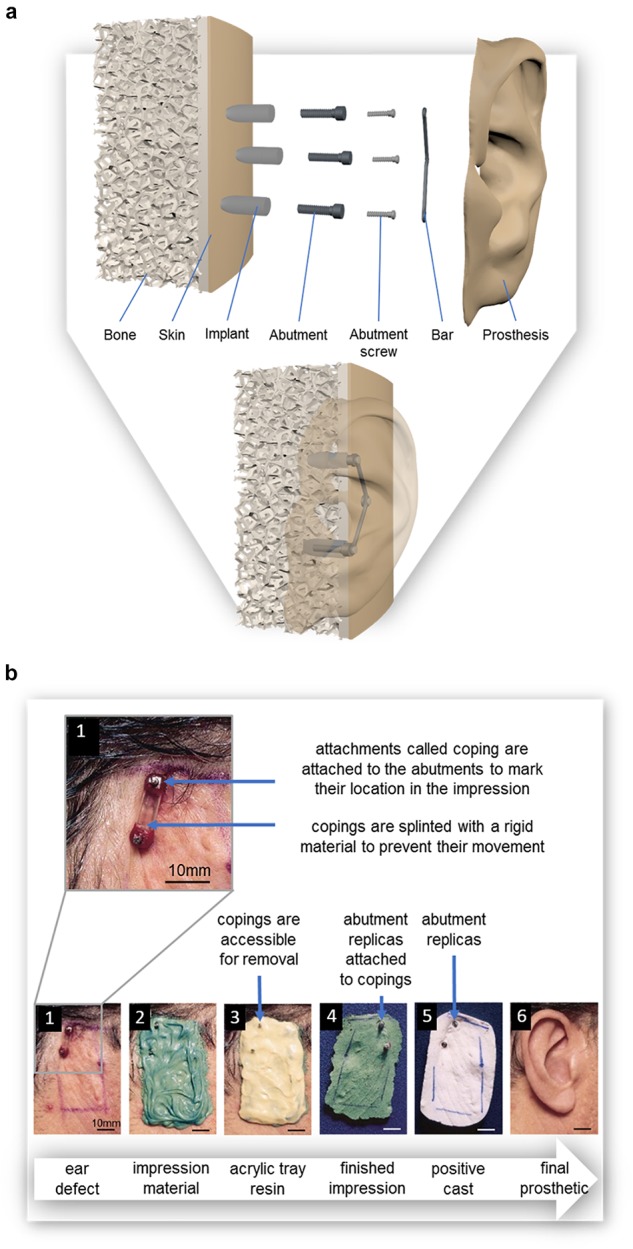
**(a)** Diagram of osseointegrated implants for the use of a bar and clip prosthetic attachment system; exploded view and assembled view, **(b)** The pick-up method for taking an impression of (1) the defect site, in this case the location of a missing ear; where attachments called impression copings are attached to the abutments prior to the (2) application of the polyvinyl siloxane impression material and are removed with (3) the custom-made impression tray; (4) Abutment replicas are attached to the impression copings after the impression is set so that they can be incorporated into (5) a positive cast of the defect on which 6) the final prosthesis is sculpted. Reproduced with permission from *Elsevier* (Kubon and Anderson, 2003).

The traditional method for fabricating a prosthesis often requires taking impressions of the existing tissue structures on the patient (Castillo and Ruiz, 2012). These structures include anatomical features and, in the case of prostheses with mechanical attachments, the location of the abutments. These abutments are connected to osseointegrated implants and are used to attach a prosthesis to the wearer through the use of a bar onto which the prosthesis may be clipped [as shown in Figure 5a or by the use of magnets (Karakoca et al., 2008)]. The impression is fundamental in ensuring passive fit of the finished prosthesis; defined as “the absence of strain development following framework fixation,” or as a 10–150 μm gap between framework and abutments (Pozzi et al., 2013). The bending moments and loading stresses of a misfit may result in damage to the prosthesis or to the patient’s bone; including loss of attachment, fracture of veneering material, screw loosening, framework fracture, screw fracture, implant fracture, bone remodeling, micro-damage, and/or loss of osseointegration (Lee et al., 2008; Pozzi et al., 2013).

**FIGURE 6:**
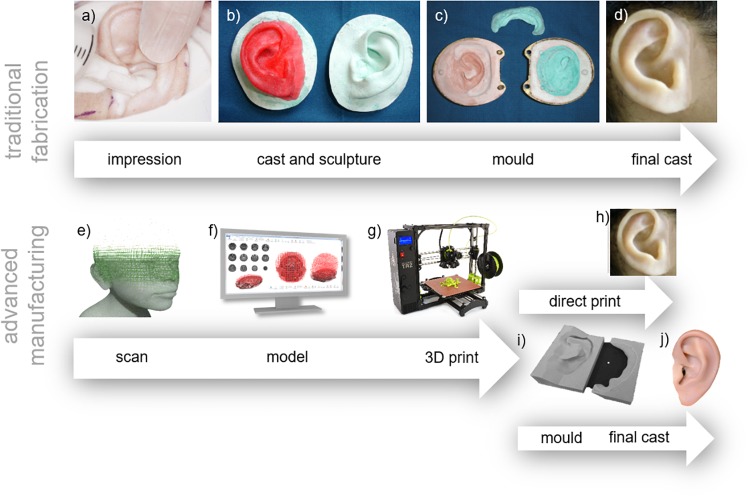
Comparison of traditional and advanced techniques. Traditional techniques follow the workflow of **(a)** impression taking - Reproduced with permission from John Wiley and Sons (Mathews et al., 2000), **(b)** casting existing structures and sculpture of the prosthesis, **(c)** creating the mould, and **(d)** final cast – Reproduced with permission from Elsevier (Subburaj et al., 2007), **(e)** 3D scanning, **(f)** 3D modeling, **(g)** 3D printing, reproduced under CC BY-SA 4.0 International by Fargo Additive Manufacturing Equipment 3D **(h)** the final prosthesis or **(i)** the mold for panel **(j)** the final cast.

The most common impression materials are polyvinyl siloxane and polyether; studies comparing these two materials have found no difference in resulting accuracy of the impression (Figure 6b; Kubon and Anderson, 2003; Lee et al., 2008; Baig, 2014). Irreversible hydrocolloid (known more commonly as dental alginate) and silicone also appear commonly as impression materials in prosthetic and dental literature (Coleman et al., 1995; Mathews et al., 2000; Kubon and Anderson, 2003; Baig, 2014). Impression material can be supported by a rigid material such as impression plaster, acrylic tray resin (Figure 6c), or wire mesh (Hutcheson and Udagama, 1980; Coleman et al., 1995; Wolfaardt and Coss, 1996; Kubon and Anderson, 2003; Karakoca et al., 2008). The use of impression plaster as a support material requires the application of a thick layer, the weight of which is known to cause distortion of the soft tissues during impression, but with minimal expansion during setting which would have caused further distortion of the impression (Coleman et al., 1995; Kubon and Anderson, 2003). Although acrylic tray resin is known to contract during polymerization distorting the impression, this is minimized by the addition of fillers (Kubon and Anderson, 2003). Acrylic support can also be relatively thin, resulting in a lightweight impression. The greatest disadvantage is the exothermic setting of acrylic which may cause discomfort and even burn the patient’s skin (Kubon and Anderson, 2003; Karakoca et al., 2008).

Impressions are preferably taken with the patient in a physiological rest position (i.e. sitting upright) to maintain accuracy, as lying down may cause distortion of the soft tissues (Kubon, 2001; Kubon and Anderson, 2003). Taking an impression may require the patient to remain still for several minutes, during which time the patient may experience discomfort or irritation from the impression material (Castillo and Ruiz, 2012).

To include the location of the abutments in the impression, temporary attachments called impression copings are placed onto the abutments, these copings may be splinted with a rigid material to prevent their individual movement while taking the impression (Figure 6a; Kubon and Anderson, 2003; Lee et al., 2008; Pozzi et al., 2013), a practice supported by most recent studies (Lee et al., 2008; Pozzi et al., 2013; Baig, 2014). Low shrinking autopolymerising acrylic resin is the most common material used for splinting (Lee et al., 2008; Pozzi et al., 2013). Alternatively, impression plaster can be used. While impression plaster sets quickly, is easy to handle, is less time consuming and less expensive than acrylic resin, it is also susceptible to fracture (Pozzi et al., 2013).

Impressions of the copings are either taken with transfer (i.e. closed tray) or pick up (i.e. open tray) methods (Kubon, 2001; Chee and Jivraj, 2006; Lee et al., 2008; Ahmed et al., 2010; Pozzi et al., 2013). In transfer methods, impressions are taken of copings and the surrounding structures with closed impression trays. The copings remain connected to the abutments as the impression is removed. In pick up methods such as that depicted in Figure 5b, impressions are taken of copings and the surrounding structures with custom made trays with openings to access the copings (Figure 5b-3). The copings can then be unscrewed and removed with the impression. Studies have indicated that pick up methods produce more accurate results with regards to the location of abutments (Chee and Jivraj, 2006; Baig, 2014).

#### Cast and Sculpture

Casts are produced by pouring plaster or dental stone into the impressions (Kubon and Anderson, 2003; Castillo and Ruiz, 2012). From these casts the prosthesis can then be sculpted by hand in dental wax using carving tools, bristle brushes and an alcohol torch (Guttal et al., 2009; Castillo and Ruiz, 2012), though sometimes clay is used. This is the most time consuming step in fabrication and the final result is highly dependent on the skills of the technician (Castillo and Ruiz, 2012). The model is sculpted from descriptions of a pre-existing structure (Castillo and Ruiz, 2012) or, in the cases where bilateral structures are present (e.g. an existing ear), by repetitive comparison with a cast of those bilateral structures, as shown in Figure 6b.

#### Mold

Molds, like those shown in Figure 6c, are then made using the wax model described above. Previously, when prostheses were made with polymers that required high temperatures to cure, metal molds were used (Guerra and Canada, 1976; May and Guerra, 1978; Oral and Zini, 1978; Choy et al., 1981; Chambers et al., 1996; Lund, 1996; Lai and Hodges, 1999; Mekayarajjananonth et al., 2003). The fabrication of these molds was difficult, expensive, and time consuming (Zini et al., 1975; Lund, 1996; Lai and Hodges, 1999); resulting in highly expensive prostheses for those with disfigurements. They were produced by first creating investment models; replicas of the wax model made with a material able to withstand high temperatures (e.g. dental stone). Then linotype metal melted to 260°C was poured onto the investment models which had been heated to 150°C (Zini et al., 1975; Guerra and Canada, 1976).

Today, plaster and dental stone (gypsum) molds are produced using the “lost wax method,” where plaster or dental stone is poured over the wax model and then the wax is simply removed with hot water (Chambers et al., 1996; Lund, 1996; Mekayarajjananonth et al., 2003; Guttal et al., 2009; Castillo and Ruiz, 2012). These molds are easy to construct and are inexpensive, while maintaining accuracy. They are, however, quite fragile and susceptible to fracture, even when reinforced (Chambers et al., 1996; Lund, 1996; Lai and Hodges, 1999; Mekayarajjananonth et al., 2003). Sometimes damage can be corrected, but often the entire mold (and wax model) must be replaced.

#### Final Cast

The inner surface of the mold can be painted and prosthetic material, often tinted silicone, is poured into the mold. After the material cures, the addition of more details such as painted skin defects and facial hair can be added to produce the final prosthesis (Karakoca et al., 2008; Guttal et al., 2009; Castillo and Ruiz, 2012).

### 3D Printing and Advanced Manufacturing

Traditional hand-crafted approaches for manufacturing prostheses are being increasingly replaced by 3D printing and advanced manufacturing approaches which are revolutionizing the way in which we can make medical devices, proving faster, cheaper and more customized solutions. Regardless of the particular prosthesis being made, 3D printing approaches utilize the same general techniques: 3D scanning of the patient’s anatomy, 3D computer modeling of the prosthesis, and either direct 3D printing of the prosthesis or printing of a mold. A visual comparison between traditional and 3D printing approaches is shown in Figure 6.

#### 3D Scanning

Scanning of anatomical structures depicted in Figure 6e can be broadly separated into clinical medical scans and 3D surface scans. Clinical scans include computed tomography (CT) (Penkner et al., 1999; Subburaj et al., 2007; Zeng et al., 2008; Liacouras et al., 2011; Qiu et al., 2011; Sun et al., 2013; Kang et al., 2016) and magnetic resonance imaging (MRI) (Kang et al., 2016). These approaches use x-rays and nuclear magnetic effects to produce 3D images of tissue structures within the body. Although capable of generating 3D models of patient specific anatomy, clinical scans are expensive, have typically low imaging resolution (several millimeters per voxel), and require the patient to lie down (causing distortion of the soft tissue surface) (Sun et al., 2013). Furthermore, use of these techniques has limited use due to radiation exposure in the case of CT scans, or dangerous interaction with the magnetic field for patients with metallic implants. However, by penetrating the tissue surface, they are able to detect the surfaces of concavities that cannot be accessed by surface scanners (Sun et al., 2013).

Another approach for obtaining 3D models of patient anatomy is 3D surface scanning. One technique, laser scanning, directs a laser onto the patient and the reflected light is used to determine 3D geometry. During scanning, the patient must not move and, in some cases, may be required to lie down (causing soft tissue deformation) to prevent any movement (Ciocca et al., 2010b). Alternatively, a physical cast (alginate or plaster) may be scanned in place of the patient (Ciocca and Scotti, 2004; Chandra et al., 2005; Watson and Hatamleh, 2014). These scans can either be taken from a stationary scanner (Coward et al., 1997, 2000; Ciocca and Scotti, 2004; Ciocca et al., 2007, 2010a,b,c; De Crescenzio et al., 2011; Reiffel et al., 2013; Watson and Hatamleh, 2014), or a hand held scanning device (Chandra et al., 2005). Complete patient scans from stationary scanners have been achieved where several scans must be taken from different angles and then aligned in post-processing (Ciocca and Scotti, 2004; Ciocca et al., 2007, 2010b,c; De Crescenzio et al., 2011). Handheld laser scanning approaches have also been developed, some with real time assembly of scans into larger 3D models. The motion of the scanners during scanning can be tracked with electromagnetic motion tracking (Chandra et al., 2005), a measurement arm (Reichinger et al., 2013), or preplaced visual markers (Reichinger et al., 2013). The use of lasers for scanning also introduces relatively high costs and eye safety hazards (Ciocca et al., 2010b, c).

Another approach is structured light scanning, where a light pattern is projected onto the patient and the reflected pattern is observed from several cameras (Wu et al., 2008; Feng et al., 2010; Sun et al., 2011, 2013; Rennesson, 2012). Here, the computer uses information about the distortion of the structured light pattern to determine distance to the surface and compute resultant 3D geometries. These scanners produce comparable resolution to laser scanners but without eye-safety concerns.

Lastly, 3D photography has been applied in producing 3D surface models of patient anatomy (Zardawi et al., 2015b). The 3dMD systems (3dMD LLC, Atlanta, GA, United States) use images taken simultaneously from cameras of known distances and angles to produce accurate 3D models (Liacouras et al., 2011; Sabol et al., 2011). The patient is only required to remain still for a short time and their eyes may be kept open. A similar approach, called photogrammetry, uses many photographs of the patient’s anatomy taken from different locations to reconstruct a 3D point cloud of significant features which are then stitched together to produce a 3D model. One advantage of photogrammetry over other scanning techniques is the ability to use accessible cameras such as those found in smart-phones (Ross et al., 2018a).

#### Computer-Aided Design

Regardless of the scanning technique used, post-processing is required; such as model alignment if there are multiple scans, elimination of abnormalities by deleting/editing mesh geometry, smoothing of bumps, scaling the scan to the correct dimensions, hole filling, and remeshing (Ciocca and Scotti, 2004; Ciocca et al., 2010b; Sun et al., 2011).

Computer-aided design (CAD), also referred to as computer modeling or 3D modeling is performed using a wide variety of existing CAD programs and software suites. After scans are obtained and converted into a polygon mesh, the software is used to produce a 3D model of the required prosthesis. In some cases, scanned patient anatomy is mirrored and forms the basis for the prosthetic computer model (Ciocca et al., 2010c; De Crescenzio et al., 2011) and in other cases, a library of anatomical models are available to be used (Wu et al., 2008; Ciocca et al., 2010a,b,c; Qiu et al., 2011; Fantini et al., 2013; Sun et al., 2013). A study by Ciocca et al. (2010c) produced a partial facial prosthesis including nose using a combination of patient and library geometry.

#### Rapid Prototyping

**FIGURE 7:**
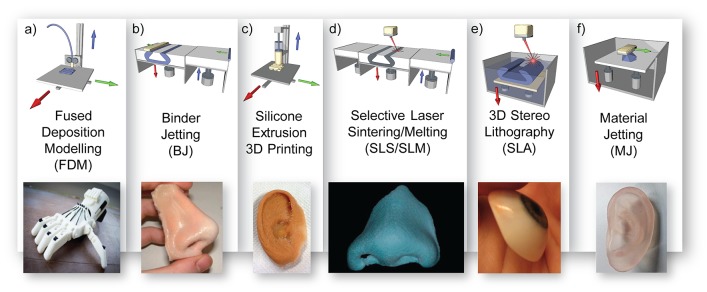
3D printing processes in prosthetics; **(a)** fused deposition modeling diagram with prosthetic hand example reproduced with permission from *Inpressco* (Mounika et al., 2017), **(b)** 3D powder printing diagram with prosthetic nose example reproduced with permission from JM Yates (Zardawi et al., 2015a), **(c)** silicone extrusion printing diagram with prosthetic ear example reproduced with permission from Fripp Design, **(d)** selective laser sintering diagram and wax nose reproduced with permission from Elsevier (Wu et al., 2008), **(e)** 3D stereolithography diagram with eye model, Creative Commons Attribution-ShareAlike by Topaz, and **(f)** material jetting diagram with ear model example reused with permission from Mohammed et al. of Deakin University.

Advanced manufacturing technologies of external prosthetics can be broadly divided into subtractive manufacturing and additive manufacturing. Subtractive manufacturing involves the use of a computer numerical controlled (CNC) mill to carve a prosthesis from a block of polymer material, such as polyurethane (Penkner et al., 1999). This process has recently given way to additive manufacturing, also referred to as 3D printing; a layer by layer manufacturing technique to produce 3D physical models from a CAD file (Rengier et al., 2010; Hofmann, 2014). Figure 7 shows several common additive manufacturing approaches that are capable of fabricating complex objects out of a large range of materials including rigid polymer models, wax models, molds, and even full prostheses.

##### Stereolithography

In the 1980s, the first 3D printing process was developed by Charles Hull, who called it stereolithography (SLA). It became commercially available in 1988 as the SLA-250 (3D Systems, Rock Hill, SC, United States) (Ventola, 2014). As illustrated in Figure 7e, SLA uses a liquid photopolymer, a combination of monomer and oligomer components contained within a vat, which is selectively cured in a layer-by-layer manner via ultraviolet (UV) crosslinking (Rengier et al., 2010; Sabol et al., 2011; Hofmann, 2014). The most common approach is to write the layer patterns using a UV laser beam, although 2D image projection methods also exist. The printing bed descends by one layer height after the cure of each layer in preparation of the next layer (Hofmann, 2014).

**FIGURE 8:**
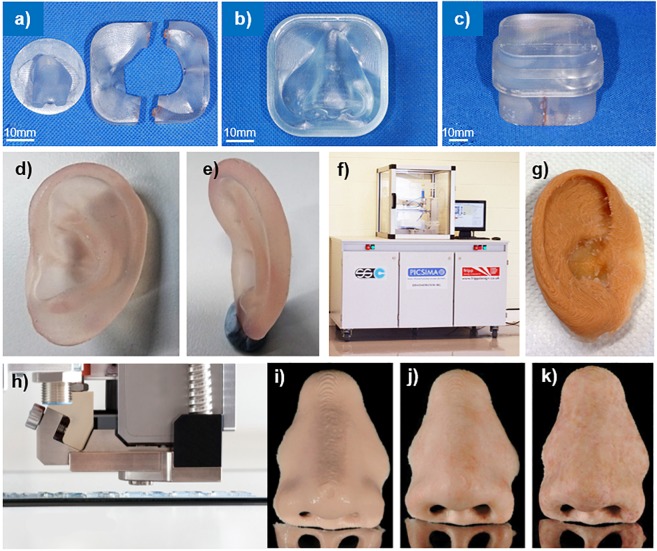
3D printed prosthetics; **(a)** parts 1, 2, and 3, **(b)** part 4, **(c)** complete prosthetic mold. Reproduced with permission from *Springer* (Qiu et al., 2011); PolyJet 3D printed ear model from panel **(d)** side, and **(e)** behind. Reproduced with permission from Dr. Mazher Iqbal Mohammed, Deakin University (Mohammed et al., 2016); **(f)** The Picsima silicone printer with **(g)** a 3D printed silicone ear. Reproduced with permission from Fripp Design. and **(h)** the Drop-on-Demand ACEO system with a 3D printed nose **(i)** without post-processing, **(j)** sealed with silicone coating and colored, and **(k)** polished with fine milling cutter, sealed with silicone coating, and colored. Reproduced with permission from *Elsevier* (Unkovskiy et al., 2018).

The advantage of SLA over other techniques is that the polymers are highly cross-linked and therefore have a strong polymer network. The use of a laser to control the patterns leads to very high printing resolution. The SLA 7000, used to produce a prosthetic model (Sabol et al., 2011), printed with a minimum layer thickness of 0.0254 mm (3D_Systems, 2019). SLA can also produce polymeric materials of varying properties including biocompatible and flexible materials (Hofmann, 2014). The materials costs are also relatively low and the process leaves little material wastage. The greatest disadvantage of SLA is potential curling and warping of the polymer. This deformation is due to the internal stresses within the structure from fast polymerization and cure shrinkage. However, by tuning the cure rate, this curling and warping can be reduced (Hofmann, 2014). The application of SLA in the fabrication of prostheses is additionally limited by the availability of photocuring materials. Currently, groups have used SLA to produce prosthetic prototypes (Sabol et al., 2011; Sun et al., 2011) and prosthetic molds (Figures 8a–c; Qiu et al., 2011) fabricated with acrylic resin.

##### Selective laser sintering

Selective laser sintering (SLS), as depicted in Figure 7d, uses thermal energy to fuse layers of powdered material. A roller first deposits a thin layer of powdered material and a laser then selectively sinters the powder into the desired pattern. With each layer of the printed object, the print bed descends and a layer of fine powder is spread over the print bed. The layer of powder is then selectively fused by a high powered infrared laser beam, sintering the powder into a solid material (Hofmann, 2014). The process of SLS requires the powdered material to be able to be sintered at high temperatures; such as thermoplastic materials, certain metal alloys, or ceramic materials (Rengier et al., 2010; Hofmann, 2014; Ventola, 2014).

SLA requires a great amount of energy to power the laser beam which serves as the energy source to fuse the material particles together, rather than a trigger for polymerization (as in SLA). Comparatively, one study (Sabol et al., 2011) employed the use of a 0.8 W SLA machine (3D_Systems, 2019) to produce a polymer prosthetic model, while another study (Feng et al., 2010) used a 30–50 W SLS machine (Yuan et al., 2016) to produce a wax prosthetic model with a minimum later thickness of 0.08 mm (Yuan et al., 2016). Consequently, SLS printers are more expensive to operate. SLS can be used to produce prostheses out of polystyrene resin (Wu et al., 2008) and PBS (polybutylene succinate) prototypes as well as wax prototypes (Wu et al., 2008; Feng et al., 2010).

##### Fused deposition modeling

Fused deposition modeling (FDM), as illustrated in Figure 7a, is a 3D printing method developed by Scott Crump in 1989 (Crump, 1992). The process melts and extrudes a thermoplastic polymer filament, the most popular materials being acrylonitrile butadiene styrene (ABS) and polylactic acid (PLA), through a heated nozzle and onto a printing bed to produce a 3D object in a layer-by-layer manner (Rengier et al., 2010; Hofmann, 2014). One of the complexities inherent to FDM printing is the need to print support structures for parts of the object that contain overhangs. Following printing, this support material must be removed and connection points to the object smoothed. Recently, the ability to print in multiple materials using multiple print heads has been commercialized. This has enabled the use of a water soluble support material which can then easily be removed with hot soapy water (Ciocca et al., 2010a,b,c; De Crescenzio et al., 2011; Sun et al., 2013). Due to the simplicity of the approach, FDM is an inexpensive and common method of 3D printing.

For 3D printing of prostheses, FDM has been used for the fabrication of low-cost prototypes (Subburaj et al., 2007; Sun et al., 2013) and molds (Ciocca et al., 2010a,b,c; De Crescenzio et al., 2011; He et al., 2014). A limitation of the extrusion method is the minimum physical thickness of each layer which can lead to a “staircase” effect due to the layer-by-layer process by which they are made; layer thickness is typically in the range of 0.3mm for low-cost (US$570) options (He et al., 2014) to 0.05 mm for more advanced printers (US$5000) (Aleph Objects, 2019). When used to produce molds for silicone casting, the staircase effect affects the surface quality and roughness of the resultant silicone prosthesis. He et al. (2014) published a method of polishing an FDM printed mold with an acetone vapor to reduce the “staircase” effect described, however this only works when an acetone-soluble material, such as ABS, is used as the mold material (He et al., 2014).

##### Material jetting

Material jetting uses inkjet (piezo electric) technology to selectively deposit liquid material in a layer by layer fashion as illustrated in Figure 7f. Following deposition, the material is solidified by a UV lamp in the case of a photocurable polymer or sets as it cools in the case of a thermoset material (e.g. wax).

One example is the Polyjet technology printer (Stratasys, Ltd., Eden Prairie, MN, United States) which deposits tiny droplets of polymer in a layer-by-layer fashion to produce the desired 3D object. With each layer, an ultraviolet lamp photocures the deposited polymer in preparation for the next layer.

One advantage of material jetting is that this method can produce 3D objects made of multiple materials with a high layer resolution of 0.1 mm (Stratasys, 2019). Multi-head MJ printers can produce complex multi-material objects and can tailor the material properties on a microscopic scale by selectively mixing materials during deposition (e.g. mixing rigid materials with flexible materials at chosen ratios to produce the desired properties). Like FDM, however, there is a need to print support structures for overhangs.

Mohammed et al. (2016, 2017) employed PolyJet technology to produce a multimaterial prosthetic ear (Figures 8d,e) and nose prototypes using the Stratasys’ Connex 3 500 3D printer. This proprietary technology is designed to only print with Stratasys’ materials; in this case, “Tango Plus,” a clear rubber like material and “Vero,” a rigid colored material. While “Tango Plus” was found to provide a soft tactile feel desirable in a prosthetic material, it was colorless. For the prosthetic ear, “Vero” was used to provide the color and rigidity to mimic cartilage tissue. The Connex printer used in this study was only capable of printing with three materials simultaneously, which prevented accurately coloring the prosthetic ear as shown in Figure 8e. Furthermore, it was found that printing thicknesses less than 1mm lead to rupture of the prosthesis during removal of the support material. To mitigate this, the model was modified to increase the thickness of or remove areas with thicknesses below 1 mm (Mohammed et al., 2016, 2017).

Alam et al. (2017) similarly, used PolyJet technology to print hollow prosthetic eyes with medical-grade acrylic resin. These were then hand-painted to match the patient’s unaffected eye. They found that the use of advanced manufacturing techniques reduced time to fabricate the prosthetic eyes from 10 to 2.5 h and resulted in superior comfort when compared with traditional methods.

A wax Thermojet Printer (3D Systems) has also been used to produce wax prosthetic prototypes (Chandra et al., 2005). These prototypes were then able to be further processed using traditional methods to produce a final prosthesis.

##### Powder printing

Powder printing (also known as binder jetting) uses inkjet technology from 2D printers to fuse layers of powdered material (e.g. plaster or starch) with a liquid resin (Rengier et al., 2010; Hofmann, 2014; Ventola, 2014; Fereshtenejad and Song, 2016). A schematic of this technique is shown in Figure 7b. With each layer of the printed object, the print bed descends and a layer of fine powder is spread over the print bed. A 2D pattern of bound powder is then produced by controlling the deposition of the liquid resin over the layer. The 3D object is then built up layer-by-layer by repeating this process (Hofmann, 2014; Fereshtenejad and Song, 2016). This solid is initially fragile (Zardawi et al., 2015b) but can be infiltrated with material such as acrylic resin (cyanoacrylate) for strength (Ciocca and Scotti, 2004; Liacouras et al., 2011; Sabol et al., 2011). One advantage of the approach is that multiple print heads can be used, each jetting a different color, enabling full color 3D printed object to be fabricated (Hofmann, 2014). Though relatively expensive compared to FDM printers, binder jetting printers such as the ZPrinters (3D_Systems, 2010) which retailed at US$ 14,900 at their least expensive (3D Systems), have been employed in the different stages of prosthetic fabrication; such as models (Ciocca and Scotti, 2004; Watson and Hatamleh, 2014) and molds (Ciocca et al., 2007; Liacouras et al., 2011; Sabol et al., 2011) with layer thicknesses as low as 0.1 mm (Liacouras et al., 2011; Sabol et al., 2011).

Due to the difficulties in directly 3D printing silicone for use in soft tissue prostheses, most studies to date have concentrated on 3D printing molds. Fripp Design (Sheffield, United Kingdom) and the University of Sheffield have bypassed the molding process and have directly 3D printed prostheses. Their initial system involved color printing onto a starch powder with a binder jetting Zprinter (3D Systems) and then infiltrating the print with medical grade silicone (Xiao et al., 2013, 2014; Zardawi et al., 2015a, b). Zardawi et al. (2015a,b) compared these 3D printed prostheses with handmade silicone polymer prostheses and found the infiltrated starch specimens had lower tensile strength, percentage elongation and tear strength. They concluded that the final 3D printed prostheses were significantly harder and had lower mechanical properties.

##### Silicone 3D printing

More recently, efforts have been made to directly print silicone prostheses. These printers have the potential to revolutionize prosthetic production, allowing the direct fabrication of realistic and customized silicone prostheses from 3D models.

In 2016, Fripp obtained a patent for a new 3D silicone printer technology, the Picsima (Figures 8f,g; Grunewald, 2016). Their patent employs room temperature vulcanizing (RTV) platinum catalyzed silicone. By selectively injecting a catalyst into a vat of the uncured silicone, a 3D silicone object can be produced.

Another attempt at direct 3D printing of silicone, described in Jindal et al. (2016, 2017), involves the development of an extrusion-based silicone 3D printer. This printer uses a two-part RTV silicone; the composition (percentage of crosslinking, filler, and catalyst as well as blend of silicone chain lengths) of which was tailored to achieve optimal mechanical properties (Jindal et al., 2016). The two silicone components are loaded into separate controlled syringe pumps mounted onto the head of the vertical axis of the printer. The components are then extruded together into a mixing device prior to being deposited onto the x-y stage. As the RTV silicone used in the printer normally cures in under one minute, a moderator was incorporated into both components to extend the working time to 30 min (Jindal et al., 2017). A thixotropic agent (a time-dependent shear thinning agent) was also added to both components to increase the viscosity of the printed silicone, thereby enabling a more rigid and stable printed structure (Jindal et al., 2017).

A recent clinical report by Unkovskiy et al. (2018) described their directly printed silicone prosthesis for a nasal defect in comparison with a traditionally fabricated prosthesis. This prosthesis was printed using the Drop-on-Demand ACEO system (Wacker Chemie AG, Munich, Germany), in which droplets of material are selectively deposited and cured with a UV lamp (Figure 8h). They found that the fit of the directly printed silicone prosthesis was clinically acceptable owing to the precision of the digital process. However, they noted that the marginal adaptation was not as smooth as with traditionally fabricated prostheses due to the layer thickness (0.4 mm) of the direct 3D printed prosthesis, requiring post-processing (Figures 8i–k).

Overcoming some of these limitations is critical for direct 3D printed silicone prosthetics. A recent rheological study by Courtial et al. (2019) found that standard silicone formulations do not provide sufficient yield stress for liquid deposition modeling 3D printing of silicone, thereby limiting their applicability (Courtial et al., 2019). To overcome this, different lengths of polyethylene glycol were added to the silicone as yield stress agents. They found this approach lead to drastic improvements without negatively impacting the final mechanical properties. Research addressing the rheological limitations of silicone were also addressed by Zhou et al. (2019), who added, nanosilica to improve their direct ink writing 3D printing (Zhou et al., 2019). This work produced a highly stretchable silicone (elongation to break of 2000%), which could be printed in high speed leading to the potential application in the direct 3D printing of robust silicone prosthetics.

Zhao et al. (2019) recently introduced photo curable approach for direct 3D printing silicone using a digital light projector (DLP) ceramic 3D printer and novel formulations of photosensitive silicone resins (Zhao et al., 2019). In this work, they produced a series of photoresins using different content of reinforcing filler silica particles and photoinitiators, resulting in DLP 3D printable silicone elastomers that have tunable mechanical properties and hardness. These silicone based elastomers, along with the other methods for direct 3D printing silicone such as extrusion-based approaches, will profoundly impact prosthetic fabrication and potentially enable directly printed prosthetics that have customizable materials properties to more precisely meet the needs of each patient.

## Conclusion

The impact of synthetic polymers on the lives of millions of people worldwide cannot be understated, with significantly improved function and aesthetics over natural materials. The challenge for materials scientists, prosthetists and technologists is to develop synthetic materials and manufacturing capabilities to enable highly personalized and life-like prosthetics that mimic the unique properties of tissue. In addition, prosthetics are worn daily over many years and need to withstand environmental conditions such as salt water, UV light, cleaning solutions, skin secretions, biological contamination and physical wear and tear. As such, it is important to understand the chemical, physical, and biological changes of polymers over their useful lifetime to ensure the soft tissue prosthetic provides optimal performance for the patient to improve their quality of life.

Although no ideal synthetic polymer yet exists, the progression of materials science has produced many impressive advancements, with better aesthetics, attachment options, fabrication techniques, material robustness and patient wellbeing. Also important is the materials choice and design for various regions of the body.

In this part A of this two part review, we discussed the history of prosthetics, desirable properties of polymeric prosthetic materials, applications of polymers in external prosthetics and fabrication methods for external prosthetics, including traditional and advanced manufacturing approaches. In part B of this review, we will detail the chemistry of commonly used, and some historical, synthetic polymers used in soft tissue prosthetics, including the polymer fundamental chemistry and synthesis, fabrication approaches, materials properties and degradation.

Modern prosthetic materials have impressive characteristics. New and upcoming advanced manufacturing and 3D printing technologies and materials will replace traditional hand-crafting approaches and revolutionize the achievable levels of realism and function of prostheses, with the goal of improving the lives of millions of people worldwide. For many conditions, tissue engineered and biofabrication approaches promise to offer an alternative to prosthetics, restoring aesthetics and function using the patient’s own tissue (Paxton et al., 2016). For example, recent studies have shown the fabrication of 3D porous ear cartilage scaffolds based on the patient’s morphology and containing the patient’s cartilage cells which were surgically implanted under the patient’s skin (Ross et al., 2018b; Zhou et al., 2018). Although there has been much recent progress in the field, significant challenges remain before biofabrication and tissue engineering is available for routine clinical use. The potential availability of tissue engineered solutions, however, may not always be suitable or desired by the patient. Future advances in soft-tissue prosthetics will emerge from close collaboration between researchers, industry, clinicians and healthcare teams, and patients, leading to better, lower cost and more robust prosthetics.

## Author Contributions

MW and SP contributed to the conception of the manuscript, structured, reviewed, and revised the manuscript. RC and MR researched and wrote the first draft and revised the manuscript. All authors contributed to manuscript revision, and have read and approved the submitted version.

## Conflict of Interest

The authors declare that the research was conducted in the absence of any commercial or financial relationships that could be construed as a potential conflict of interest.
